# Psychological Impact of the COVID-19 Pandemic and Social Determinants on the Portuguese Population: Protocol for a Web-Based Cross-sectional Study

**DOI:** 10.2196/28071

**Published:** 2021-10-19

**Authors:** A Aguiar, M Pinto, R Duarte

**Affiliations:** 1 EPIUnit Instituto de Saúde Pública Universidade do Porto Porto Portugal; 2 Laboratório para a Investigação Integrativa e Translacional em Saúde Populacional (ITR) Porto Portugal; 3 Faculdade de Psicologia e Ciências da Educação Universidade do Porto Porto Portugal; 4 Unidade de Investigação Clinica da ARS Norte Administração Regional De Saúde Do Norte Porto Portugal; 5 Instituto de Ciências Biomédicas Abel Salazar ICBAS-UP Universidade do Porto Porto Portugal; 6 Serviço de Pneumologia Centro Hospitalar de Vila Nova de Gaia/Espinho Vila Nova de Gaia Portugal

**Keywords:** COVID-19, public health, mental health, study protocol, psychological impact, anxiety, depression, grief, behavior change

## Abstract

**Background:**

The COVID-19 outbreak and consequent physical distance measures implemented worldwide have caused significant stress, anxiety, and mental health implications among the general population. Unemployment, working from home, and day-to-day changes may lead to a greater risk of poor mental health outcomes.

**Objective:**

This paper describes the protocol for a web-based cross-sectional study that aims to address the impact of the COVID-19 pandemic on mental health.

**Methods:**

Individuals from the general population aged 18 years or more and living in Portugal were included in this study. Data collection took place between November 10, 2020, and February 10, 2021. An exponential, nondiscriminative, snowball sampling method was applied to recruit participants. A web-based survey was developed and shared on social media platforms (eg, Facebook, Instagram, Twitter, LinkedIn, and WhatsApp groups) and through e-mail lists for recruitment of the seeds.

**Results:**

Data analysis will be performed in accordance with the different variables and outcomes of interest by using quantitative methods, qualitative methods, or mixed methods, as applicable. A total of 929 individuals had completed the web-based survey during the 3-month period; thus, our final sample comprised 929 participants. Results of the survey will be disseminated in national and international scientific journals in 2021-2022.

**Conclusions:**

We believe that the findings of this study will have broad implications for understanding the psychological impact of the COVID-19 pandemic on Portuguese residents, as well as aspects related to the informal economy. We also hope that the findings of this study are able to provide insights and guidelines for the Portuguese government to implement action. Finally, we expect this protocol to provide a roadmap for other countries and researchers that would like to implement a similar questionnaire considering the related conclusions.

**International Registered Report Identifier (IRRID):**

DERR1-10.2196/28071

## Introduction

### Background

Mental health is an integral and essential component of health. It constitutes a relevant and increasing burden of disease worldwide [1], with major depression expected to be the largest contributor by 2030 [2]. Thus, mental health is extremely important, not only due to its own value for living but also because it is a critical determinant of physical health [3].

Nevertheless, mental health represents a complex public health challenge wherein environmental factors play a fundamental role. In particular, the relationship between viral illnesses and mental health conditions, especially anxiety and depression, have long been studied. Previous studies on epidemics, such as influenza [4,5], varicella-zoster [6,7], herpes simplex, HIV/AIDS, and hepatitis C [8-11], have already described the strong links between infectious diseases and mental health outcomes.

Pandemic disasters have been part of the human history for centuries, and the human response to the COVID-19 pandemic inevitably differs from that toward other disasters, as social gatherings are being discouraged. Instead, the exact opposite—separation, isolation and quarantine—is required to manage the outbreaks [12]. Although such disease containment measures may suppress the outbreaks, they have the unintended consequences of constraining family rituals, norms, and values, which regulate and protect family functioning in times of crisis [13]. The COVID-19 pandemic and the resulting physical distancing measures implemented by many countries caused disruptions to daily routines that may have a strong impact on mental health.

In a recent Kaiser Family Foundation Health tracking poll, nearly half of adults in the United States reported that their mental health has been negatively impacted due to worry and stress over the outbreak [14]. Although necessary to prevent loss of life due to COVID-19, public health measures expose many people to situations that may be linked to psychosocial problems, such as isolation and unemployment. Additionally, anxiety and depression are increasingly common [13], as people are fearful of themselves or their loved ones falling ill, and they are uncertain of the repercussions of the pandemic, which may be linked to a greater vulnerability toward the virus [13].

Over the last 20 years, other outbreaks of infectious diseases have occurred worldwide. The most recent examples include the outbreak of severe acute respiratory syndrome (SARS) in 2002 [15] and the 2009-2010 influenza A/H1N1 influenza pandemic [16]. Profound psychosocial implications at the individual, community, and international levels have been documented in the past epidemics and pandemics [17]. However, the world has not experienced an epidemic or pandemic with the same intensity and duration as that of COVID-19 in recent times.

Therefore, the COVID-19 pandemic may have serious implications for individual and collective health, as well as emotional and social functioning in the present and future. According to a 2020 review conducted by Brooks et al [18], being forced to stay at home leads to negative psychological effects such as fear, frustration, and anger. The negative impact of confinement can have long-lasting effects on an individual. Further understanding of the biopsychosocial consequences of pandemic disasters, such as COVID-19, is the first step toward achieving best practices on global, regional, and local preparedness and response [19]. In order to understand the consequences of the COVID-19 pandemic on people’s mental health, we have two main aims under the construct of this protocol: The first aim is to explore the mental health status of the general adult population during the COVID-19 outbreak, in terms of psychological impact caused by the pandemic and resulting anxiety and depression symptoms. The second aim is to examine the extent to which different sociodemographic and other variables are associated with psychological impact, anxiety, and depression.

We believe there is an urgent need to broaden our knowledge about mental health in the Portuguese population as a first step to develop psychological interventions, so that the lasting psychological negative consequences of the pandemic can be reduced. To address this gap in the literature, this article presents the study protocol for a survey on the psychological impact the COVID-19 crisis has had on the psychological health of the Portuguese population.

### Objectives and Aims

The overall objective of this cross-sectional study is to evaluate the impact of the COVID-19 pandemic on the mental health of individuals residing in Portugal. This study is of particular importance due to the possible psychosocial impact of the COVID-19 pandemic and associated confinement measures on the mental health of individuals, as well as to examine associated socioenvironmental determinants. In particular, our research will attempt to verify and measure whether the following issues are interconnected: (1) employment status during the COVID-19 pandemic; (2) the psychological impact of online education at home on children, adolescents, and parents; (3) household food insecurity; (4) the impact of having a confirmed COVID-19 diagnosis on the individual’s mental health; (5) anxiety and depression in relation with employment status and confinement measures; (6) understanding how the pandemic may cause changes in the experiences of loss and grief; and finally, (7) domestic violence during the lockdown.

Most studies published since the beginning of the COVID-19 pandemic have focused on a set of social and mental health determinants directly related to the pandemic, in addition to epidemiological data associated with the infection. Thus, the choice of the determinants presented above is related to the important documentation and evidence that has been published in recent months, namely on the issues of unemployment due to lockdown and restrictive measures [20,21]; working from home along with children participating in online education from home [22]; food insecurity [23,24]; and the impact of the COVID-19 pandemic on mental health [25], anxiety and depression [26-28], grief [29-31], and domestic violence [32-34].

The proposed project is grounded on the importance of mapping the most relevant psychosocial mental health determinants during the COVID-19 pandemic among Portuguese residents. We expect to provide evidence based-knowledge to guide policy-level practices directly towards the reduction of health inequities, increased access to psychological services, and also raise awareness for negative mental health outcomes as a result of job loss, changes in routine, loss of someone close, and food insecurity.

The specific research aims outlined below will help us gather data for the study, including data from all subregions of the country. We aim to analyze the unique patterns of the impact of the COVID-19 pandemic through the use of a web-based survey applied to a nonprobabilistic sample of adult Portuguese residents.

#### Aim 1

We aim to analyze possible changes in employment status since the beginning of the pandemic, taking into consideration at least age, gender, occupation, and education as potential confounders. Questions on occupation before and since the beginning of the pandemic, changes in income, and pandemic-related unemployment will also be asked to fully access possible significant associations.

#### Aim 2

We aim to understand how parents and/or caregivers at home have changed their normal routines due to web-based education and working from home, and to examine the possible existing association with stress and anxiety disorders. Additionally, we will evaluate the increase in the use of anxiolytics, as reported by the National Authority for Medication and Health Products (Infarmed in Portuguese) [35].

#### Aim 3

We aim to assess the prevalence of household food insecurity among the Portuguese population since the beginning of the COVID-19 pandemic. Food security status will be evaluated using the US Household Food Security Survey Module: Six-Item Short Form [36,37]. Participants will be asked about the food eaten in their households, in the previous 12 months, and whether they were able to afford the food they need. We will also include an option for open text for participants to comment on their experiences and perceptions of food insecurity changes since the beginning of the COVID-19 pandemic in order to support their answers.

#### Aim 4

We aim to analyze increase in anxiety and depression in levels relation with COVID-19 pandemic. Anxiety and depression symptoms will be evaluated using the Hospital Anxiety and Depression Scale (HADS) [38,39], which indicates how an individual has felt in the past week. Mental health conditions, such as anxiety and depression, can affect an individual´s thought process, feelings, mood, and behavior in such a way that they can influence the individual’s ability to relate to others and to complete everyday tasks. Since these conditions can be situational—short-term or long lasting, chronic—we want to assess and understand how the COVID-19 pandemic may influence mental status.

#### Aim 5

We aim to evaluate prolonged grief disorder since the beginning of the COVID-19 pandemic. For this purpose, we will use the PG-13 Prolonged Grief Disorder as an instrument to collect and analyze data. The following criteria must be met for prolonged grief disorder to be considered: “the experience of loss-generating intense longing and yearning for the deceased must extend for at least six months” [40,41]. Based on these criteria and the fact that the first case of COVID-19 in Portugal was on March 2, 2020, and that our questionnaire was launched on November 10, 2020, those participants that did not meet these criteria will be excluded from this part of the analysis.

Besides facing arduous conditions over the last days of life due to COVID-19 protection measures, these criteria also created a challenging post-death scenario, since funerals and burials were postponed or held remotely [42,43]. Accordingly, the presence of family members and loved ones during such crucial moments became impossible. Therefore, the impact of these circumstances on the mental health of family members, as well as the anxiety and stress experienced, must be carefully examined and understood.

#### Aim 6

We aim to evaluate domestic violence between couples before and since the beginning of the COVID-19 pandemic. For this purpose, we will use an adaptation of the screening questions proposed by the Portuguese Association for Victim Support [44].

Sociodemographic characteristics were collected at the beginning of the survey and all of the measuring instruments indicated above have been previously validated for the Portuguese population.

## Methods

### Design

The study design can be characterized as a web-based cross-sectional survey and is based on the construct of the snowball sampling method. Snowball sampling began as a method for sampling networks. Coleman was a pioneer in using this technique as a method for studying a person’s social environment [45]. Later, Goodman and Kish presented a more rigorous version of the method using a probability sample [46].

The snowball sampling can be defined as “[A] technique for finding research subjects. One subject gives the researcher the name of another subject, who in turn provides the name of a third, and so on. This strategy can be viewed as a response to overcoming the problems associated with sampling concealed hard to reach populations such as the criminal and the isolated” [47]. In our study, a virtual snowball sampling survey was disseminated first through social networking channels, namely Facebook, Instagram, LinkedIn, WhatsApp groups, and Twitter, and then by using the personal mailing lists of the researchers involved.

The selection of this method for the dissemination of the questionnaire was based on the convenience of reaching participants from a variety of places, which increases the sample size and reduces the costs and time associated with data collection. We believe that the use of Facebook, Instagram, Twitter, LinkedIn, and personal mailing lists (all free of charge) to contact individuals can minimize problems associated with “spam” messages, impersonal contact, unclear answers, and low response rates. Moreover, the possibility of having access to *offline contacts* through the recommendation by *online contacts* can reduce problems associated with selection bias and representation.

As Brickman-Bhutta explained, “Facebook and other social network sites allow us to carry chain-referral methods into the age of the Internet, while also exploiting the strengths of online questionnaires” [48].

Therefore, in this study, participants were asked, at the beginning and at the end of the questionnaire, to share the survey with at least five of their personal contacts to ensure that the snowball sampling method was effective.

### Study Population

Participants from the general population in Portugal were enrolled in this study. The inclusion criteria were as follows: (1) age 18 years or older and (2) a resident of Portugal. Participants not meeting these inclusion criteria were excluded from the study. Participants’ birth dates will be recorded as age in years, and those aged under 18 years will be excluded from the analysis. In addition, participants who reported they live outside Portugal were excluded from the final sample.

### Study Procedures and Data Collection

**Figure 1:**
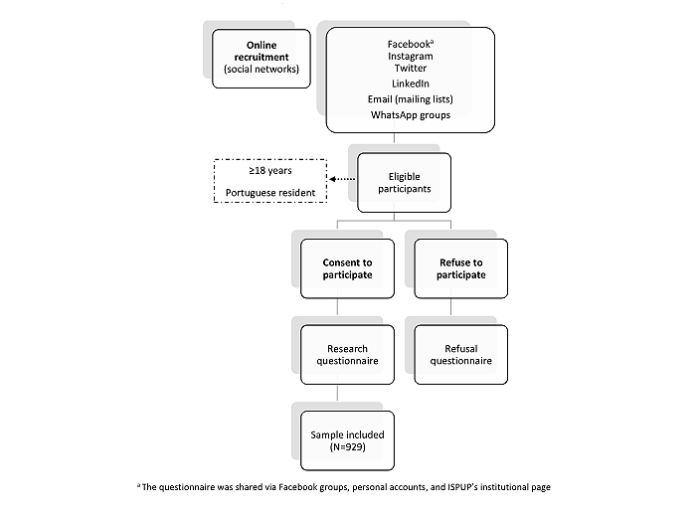
Flowchart of the study procedures and enrollment of participants. ISPUP: Instituto de Saúde Pública, Universidade do Porto.

Study procedures are illustrated in Figure 1. The enrollment was ongoing for 3 consecutive months from November 10, 2020, to February 10, 2021.

Data collection included general demographic variables, such as age, gender, region, educational level, and marital status. It also included variables related to COVID-19, such as incidence in family. In addition, information on other health-related risk factors such as a history of noncommunicable diseases was collected. To complement and to address our study aims, the survey included the evaluation of the following dimensions: (1) employment status, (2) children and web-based education, (3) food insecurity, (4) SARS-CoV-2 infection and mental health, (5) anxiety and depression, (6) grief and mourning, and (7) domestic violence.

All the instruments used to evaluate anxiety and depression, grief and mourning, and domestic violence have previously been validated for use for the Portuguese population [38,41,44]. Despite the scale used for food insecurity assessment not being fully validated for the Portuguese population, previous studies among Portuguese individuals have reported good internal consistency [49,50].

The questionnaire was administered in Portuguese—the official language of Portugal. A pretest of the questionnaire was conducted with 15 people. We asked these participants to complete the survey the same way that it would be completed in the actual project—that is, by using a web-based tool. We asked them to annotate doubts, questions, and bugs in the web-based questionnaire and possible improvements or clarifications. Thereafter, we took all the comments and improved the questionnaire by clarifying some questions and correcting typographical errors. Moreover, since the scales used to measure food security, anxiety and depression symptoms, prolonged grief disorder, and violence were already translated and validated previously for the Portuguese population, no pre-test on the translation was conducted; this was because the tools used were already reliable and have shown good internal consistency in the validation studies previously conducted.

For the purpose of possible replication of this method, an English-translated version of the questionnaire is provided in Multimedia Appendix 1.

### Data Analysis

Data will be analyzed using quantitative methods, qualitative methods, or mixed methods depending on the different variables and outcomes as described above.

#### Statistical Analysis

Characteristics of participants will be described by absolute and relative frequencies and compared using the Chi-square test or Fisher exact test, as appropriate, in categorical variables. Medians and percentiles (P25-P75) will be used to describe continuous variables compared using the Mann-Whitney *U* test. Unconditional logistic regression models will be computed to assess the associations between the instruments used and sociodemographic variables. Odds ratios and corresponding 95% CIs will be estimated.

Exploratory factor analysis using principal component analysis will be applied to the anxiety and depression scale and the PG-13 instrument. Additionally, the oblique rotation will be performed to examine the factor structure of the scales. Internal consistency of the tools will also be assessed by measuring inter-item correlation and Cronbach coefficient.

Furthermore, poststratification survey weights will be calculated to try to correct oversampled subpopulations (eg, younger, more educated participants) and undersampled subpopulations (eg, older, less educated, and male participants) [51]. Adjusting the weights will increase the stratum variance and, thereby, the design effect. We will compare our survey data with existing census for the country (2011 or 2021 census, that are being currently updated).

#### Qualitative Data Analysis

Open-ended questions will be analyzed using content analysis. In the present study, and taking into consideration the different objectives presented above, we included open-ended questions in all different sections of the questionnaire to comprehensively explore participants’ feelings, constrains, advantages, disadvantages, problems, among other aspects. Unlike a closed-ended question that leaves survey responses limited and narrowed to the given options, open-ended question allows participants to probe deep into their answers, providing valuable information about the subject at hand. The responses to these questions can be used to attain detailed and descriptive information on a topic.

The following open-ended questions were added to the survey: (1) relation with children and school from home, including changes in routine and its positive and/or negative effect on personal and professional life; (2) food security, including worsening of the household situation (eg, less money to buy food due to the COVID-19 pandemic); (3) health care, including the use of anxiolytics and/or antidepressants to explore the reasons behind starting to consume such medicines since the beginning of the COVID-19 pandemic; (4) COVID-19 infection, including the presence of anxiety or depressive symptoms in consequence of a positive diagnosis; (5) mental health, including anxiety and depression symptoms in relation with the pandemic situation; and (6) grief and mourning, including impact of the loss on one’s daily life.

Content analysis is a research tool used to determine the presence of certain words, themes, or concepts within qualitative data, such as text. By using content analysis, we will be able to explore the underlying meanings and psychosocial processes of the quantitative data collected. Thus, it will provide additional knowledge and a deeper understanding of the psychological impact of the COVID-19 pandemic [52]. We based the process of coding and analysis on the principles proposed by Braun and Clarke [53], which implied an iterative process of systematically identifying and organizing patterns of meaning (ie, themes) in the data set. An inductive coding framework was applied using a group-up approach where codes were derived from the data. A 4-step approach was performed: (1) first read of the answers and coding, (2) organization of the codes into categories and subcodes, (3) further rounds of codes whenever needed, and (4) turning the codes and categories into the final narrative.

#### Mixed Methods Analysis

The term “mixed methods” refers to an emergent methodology of research that advances the systematic integration, or “mixing” of quantitative and qualitative data within a single investigation or sustained program of inquiry. The basic premise of this methodology is that such integration permits a more complete and synergistic usage of data than separate quantitative and qualitative data collection and analysis. Mixed methods are especially useful in understanding contradictions between quantitative results and qualitative findings [54].

Considering the above-mentioned explanation, we also aim to use both quantitative and qualitative data, whenever possible. Since our open-ended questions complement some of the quantitative questions, we aim to explain the quantitative data in more detail through the qualitative data, namely data on the psychological and routine changes impact of teleschool and teleworking—the balance between work, parenting, and house care; food security changes concerning, for instance, job loss due to the pandemic and its relation with household income and affordability to buy food; use of anxiolytics and/or antidepressants due to changes in daily life concerning the pandemic—feelings of anxiety and depression; symptoms of anxiety and depression before and since the beginning of the pandemic; and exacerbated symptoms of grief and mourning due to the pandemic restrictions on proper farewell moments. Thus, the qualitative approach used in this study will only apply to some of the subset aims in order to produce deeper knowledge on the research areas of interest.

### Ethics and Informed Consent

Ethical approval was obtained from the Ethics Committee of the Institute of Public Health of the University of Porto (CE20166). All participants, by accessing the questionnaire through the link, were asked to provide informed consent according to the Ethical Principles for Medical Research involving human subjects, expressed in the Declaration of Helsinki and the current national legislation. Since this is a web-based survey, participants could select between two options: accept to participate in the study or decline to participate. For the latter, a refusal questionnaire with questions on gender, age, number of years of education completed, and the reason for not accepting to participate could be answered. The questionnaire is confidential, and no data that would allow for the identification of the participants is collected.

## Results

Participant enrollment has now been completed. A total of 929 participants completed the survey. The majority of participants were female (659/929, 70.9%). The mean age of participants was 36.8 (SD 11.5) years; a majority of the participants have a university degree (700/929, 75.3%), and 21.1% (196/929) have at least one comorbidity, and 4.4% (41/929) have had a confirmed COVID-19 diagnosis. Moreover, Sociodemographic characteristics and information on confirmed COVID-19 diagnosis of participants who were included are shown in Table 1. Results of the survey will be disseminated in national and international scientific journals in 2021-2022.

**Table 1:** Preliminary sociodemographic characteristics and COVID-19 diagnosis of participants enrolled in the study (N=929).

Characteristic		Participants, n (%)
**Gender**		
	Male	265 (28.5)
	Female	659 (70.9)
	Missing	5 (0.5)
Age, mean (SD)		36.8 (11.5)
**Education**		
	≤12 years	217 (23.9)
	University	700 (75.3)
	Missing	12 (1.3)
**Marital status**		
	Single	450 (48.4)
	Married	405 (43.6)
	Divorced	65 (7)
	Widowed	5 (0.5)
	Missing	3 (0.3)
**Parish of residence**		
	North	590 (63.5)
	Centre	98 (10.5)
	Alentejo	11 (1.2)
	Lisbon Metropolitan Area	186 (20.0)
	Algarve	18 (1.9)
	Islands (Azores and Madeira)	8 (0.9)
	Missing	18 (1.9)
**Household size**		
	1 person	144 (15.5)
	2 persons	280 (30.1)
	≥3 persons	487 (52.4)
	Missing	18 (1.9)
**Working status during the COVID-19 pandemic**		
	Continued employed	655 (70.5)
	Become unemployed	73 (7.9)
	Continued unemployed	45 (4.8)
	Student	48 (5.2)
	Housewife	5 (0.5)
	Retired	14 (1.5)
	Missing	89 (9.6)
**History of previously diagnosed diseases**		
	Yes	196 (21.1)
	No	719 (77.4)
	Missing	14 (1.5)
**Positive COVID-19 diagnosis**		
	Yes	41 (4.4)
	No	878 (94.5)
	Missing	10 (1.6)

## Discussion

### Overview

There are well-established associations between infectious diseases and mental health outcomes that have shown profound psychosocial implications at the individual, community, and international levels. The human response to pandemics such as the COVID-19 discourage social gatherings of people and warrant separation, isolation, and quarantine. Although such disease containment measures may quell the outbreak, they have the unintended consequences of constraining family rituals, norms, job loss, work from home with children to help at the same time, changes in food behaviors, and anxiety and depression.

The uncertainty of how this novel disease will develop together with the unusual situation of being confined at home is most likely leading people to experience negative psychological consequences [18]. Despite the urgent need claimed by several studies [18,55-57] to systematically examine the psychological health of the population being most affected by the COVID-19 pandemic, scientific data on this matter, concerning the Portuguese population, is still scarce. Our proposed study protocol aims to address this gap in the literature, by conducting a survey to evaluate the psychological impact of the COVID-19 crisis on the Portuguese population. Specifically, we collected data on the psychological impact of the COVID-19 crisis on the mental health of adults, including the psychological impact as well as anxiety and depression symptoms.

Findings from this study will provide useful information regarding the impact of the COVID-19 pandemic on mental health. This study will also highlight other health-related risks according to sex, education, employment status, and food security status. It also serves as an important descriptive starting point for future follow-up surveys in specific target groups.

This study will also contribute to developing national and European evidence-informed policies that translate research into effective health strategies, which are sustainable over time. According to socioeconomic dimensions, a comprehensive analysis will also contribute to developing policies that affect equity and human well-being.

### Limitations

Some limitations can be anticipated since this study comprises a nonrepresentative sample of the Portuguese population. One of the main limitations of our study is related to selection bias. Selection bias in this sample can be discussed since only specific groups of the population use the internet and social networks, such as Facebook and LinkedIn. Nevertheless, we believe that the sampling method used, despite its limitations, is still very effective and more so nowadays, given the social distancing measures imposed by the governments.

Additionally, we must consider that virtual snowball sampling techniques imply a semirandom selection procedure, which means that we cannot generalize our results for the general population. Nonprobabilistic sampling can disproportionately affect prevalence estimates in our results. Therefore, poststratified weights will be applied to make the sample more representative of the general population. Poststratification relies on the data obtained in the survey itself that were not available before sampling, and it adjusts the weights so that the totals of each group are equal to the known population total. Nevertheless, this study protocol and its preliminary results have described an efficient method that has the capability of extending the sample size, improving response rate, and recruitment effectiveness.

### Strengths

We consider that the structure of web-based questionnaires can reduce the possibility of errors because (1) it is possible to program specific instructions for each question (eg, multiple answer options, one answer only, open-ended questions, Likert scales); (2) the answers are easily visible when displayed on computers or smartphones; and (3) automatic filters can be applied for the questions, according to the respondents’ answers. Moreover, another strength of the study is the open-ended questions that permit the respondent to have the liberty to include details about their feelings, attitudes, and views that they usually would not be able to submit via responses to close-ended questions. Allowing the respondents to answer in their own words can lead to empowering outcomes. By using open-ended questions, participants are able to express and articulate opinions that may be extreme, unusual, or simply ones that the researcher did not think about when creating the survey. This often provides researchers rich, relevant data for their studies.

It is expected that the structure and information derived from this survey could also contribute to the development and consolidation of solid infrastructures for epidemiological and public health research by building a future national functioning surveillance system that can be reproducible over time. We also expect that this protocol may provide resources for future implementation in other study settings in different countries.

### Conclusions

Our findings can help in the design of group-specific national interventions so that people who have seen their psychological health diminished during the pandemic can better cope with this difficult situation, both in Portugal and other parts of the world. Considering that this current health crisis will most likely have long-lasting effects, follow-up studies are needed to obtain a clear picture of the magnitude of the psychological impact of the COVID-19 pandemic.

## Appendix

Multimedia Appendix 1 Study questionnaire translated in English.

## Abbreviations

HADS: Hospital Anxiety and Depression Scale
SARS: severe acute respiratory syndrome

## References

[1] (2001). The World Health Report 2001: Mental Health: New Understanding, New Hope.

[2] (2008). The Global Burden of Disease: 2004 Update.

[3] (2016). Editorial: What can public health do for mental health?. The Lancet.

[4] Cowling BJ, Ng DMW, Ip DKM, Liao Q, Lam WWT, Wu JT, Lau JTF, Griffiths SM, Fielding R (2010). Community psychological and behavioral responses through the first wave of the 2009 influenza A(H1N1) pandemic in Hong Kong. J Infect Dis.

[5] Miller GE, Cohen S, Pressman S, Barkin A, Rabin BS, Treanor JJ (2004). Psychological stress and antibody response to influenza vaccination: when is the critical period for stress, and how does it get inside the body?. Psychosom Med.

[6] Gilden DH, Kleinschmidt-DeMasters BK, LaGuardia JJ, Mahalingam R, Cohrs RJ (2000). Neurologic complications of the reactivation of varicella-zoster virus. N Engl J Med.

[7] Irwin MR, Levin MJ, Carrillo C, Olmstead R, Lucko A, Lang N, Caulfield MJ, Weinberg A, Chan ISF, Clair J, Smith JG, Marchese RD, Williams HM, Beck DJ, McCook PT, Johnson G, Oxman MN (2011). Major depressive disorder and immunity to varicella-zoster virus in the elderly. Brain Behav Immun.

[8] Katz S, Nevid JS (2005). Risk factors associated with posttraumatic stress disorder symptomatology in HIV-infected women. AIDS Patient Care STDS.

[9] Khan MR, Kaufman JS, Pence BW, Gaynes BN, Adimora AA, Weir SS, Miller WC (2009). Depression, sexually transmitted infection, and sexual risk behavior among young adults in the United States. Arch Pediatr Adolesc Med.

[10] Martin L, Kagee A (2011). Lifetime and HIV-related PTSD among persons recently diagnosed with HIV. AIDS Behav.

[11] Vivithanaporn P, Nelles K, DeBlock L, Newman SC, Gill MJ, Power C (2012). Hepatitis C virus co-infection increases neurocognitive impairment severity and risk of death in treated HIV/AIDS. J Neurol Sci.

[12] Sprang G, Silman M (2013). Posttraumatic stress disorder in parents and youth after health-related disasters. Disaster Med Public Health Prep.

[13] Fiese BH, Spagnola M, Masten AS (2007). The interior life of the family: Looking from the inside out and the outside in. Multilevel Dynamics in Developmental Psychopathology. Pathways to the Future: The Minnesota Symposia on Child Psychology.

[14] Panchal N, Kamal R, Orgera K, Cox C, Garfiled R (2021). The implications of COVID-19 for mental health and substance use. The Kaiser Family Foundation.

[15] Severe Acute Respiratory Syndrome (SARS). World Health Organization.

[16] 2009 H1N1 Pandemic (H1N1pdm09 virus). Centers for Disease Control and Prevention.

[17] Preti E, Di Mattei V, Perego G, Ferrari F, Mazzetti M, Taranto P, Di Pierro R, Madeddu F, Calati R (2020). The psychological impact of epidemic and pandemic outbreaks on healthcare workers: rapid review of the evidence. Curr Psychiatry Rep.

[18] Brooks SK, Webster RK, Smith LE, Woodland L, Wessely S, Greenberg N, Rubin GJ (2020). The psychological impact of quarantine and how to reduce it: rapid review of the evidence. Lancet.

[19] (2020). Mental health and psychosocial considerations during the COVID-19 outbreak. World Health Organization.

[20] Banks J, Karjalainen H, Propper C (2020). Recessions and health: The long-term health consequences of responses to the coronavirus. Fisc Stud.

[21] Kim AT, Kim C, Tuttle SE, Zhang Y (2021). COVID-19 and the decline in Asian American employment. Res Soc Stratif Mobil.

[22] Van Lancker W, Parolin Z (2020). COVID-19, school closures, and child poverty: a social crisis in the making. Lancet Public Health.

[23] Gundersen C, Hake M, Dewey A, Engelhard E Food insecurity during COVID-19. Appl Econ Perspect Policy..

[24] Wolfson JA, Leung CW (2020). Food insecurity during COVID-19: an acute crisis with long-term health implications. Am J Public Health.

[25] Xiong J, Lipsitz O, Nasri F, Lui LMW, Gill H, Phan L, Chen-Li D, Iacobucci M, Ho R, Majeed A, McIntyre RS (2020). Impact of COVID-19 pandemic on mental health in the general population: a systematic review. J Affect Disord.

[26] Choi EPH, Hui BPH, Wan EYF (2020). Depression and anxiety in Hong Kong during COVID-19. Int J Environ Res Public Health.

[27] Ozamiz-Etxebarria N, Dosil-Santamaria M, Picaza-Gorrochategui M, Idoiaga-Mondragon N (2020). Levels of stress, anxiety and depression in the first phase of the COVID-19 outbreak in a sample collected in northern Spain. Article in Spanish. Cad Saúde Pública.

[28] Stein MB (2020). Editorial: COVID-19 and anxiety and depression in 2020. Depress Anxiety.

[29] Mayland CR, Harding AJE, Preston N, Payne S (2020). Supporting adults bereaved through COVID-19: a rapid review of the impact of previous pandemics on grief and bereavement. J Pain Symptom Manage.

[30] Wallace CL, Wladkowski SP, Gibson A, White P (2020). Grief during the COVID-19 pandemic: considerations for palliative care providers. J Pain Symptom Manage.

[31] Zhai Y, Du X (2020). Loss and grief amidst COVID-19: a path to adaptation and resilience. Brain Behav Immun.

[32] Ghoshal R (2020). Twin public health emergencies: Covid-19 and domestic violence. Indian J Med Ethics.

[33] Mazza M, Marano G, Lai C, Janiri L, Sani G (2020). Danger in danger: Interpersonal violence during COVID-19 quarantine. Psychiatry Res.

[34] Neil J Domestic violence and COVID-19: our hidden epidemic. Aust J Gen Pract. Epub ahead of print posted online on June 11, 2020.

[35] Diário DN (2020). Em três meses, vendidas mais de 5 milhões de embalagens de ansiolíticos e antidepressivos. Article in Portuguese. Diário de Notícias.

[36] (2012). U.S. Household Food Security Survey Module: Six-Item Short Form. United States Department of Agriculture, Economic Research Service.

[37] Blumberg SJ, Bialostosky K, Hamilton WL, Briefel RR (1999). The effectiveness of a short form of the Household Food Security Scale. Am J Public Health.

[38] Pais-Ribeiro J, Silva I, Ferreira T, Martins A, Meneses R, Baltar M (2007). Validation study of a Portuguese version of the Hospital Anxiety and Depression Scale. Psychol Health Med.

[39] Zigmond AS, Snaith RP (1983). The hospital anxiety and depression scale. Acta Psychiatr Scand.

[40] Prigerson H, Horowitz M, Jacobs S, Parkes C, Aslan M, Goodkin K, Raphael B, Marwit Samuel J, Wortman Camille, Neimeyer Robert A, Bonanno George A, Bonanno George, Block Susan D, Kissane David, Boelen Paul, Maercker Andreas, Litz Brett T, Johnson Jeffrey G, First Michael B, Maciejewski Paul K (2009). Prolonged grief disorder: psychometric validation of criteria proposed for DSM-V and ICD-11. PLoS Med.

[41] Delalibera M, Coelho A, Barbosa A (2011). Validation of prolonged grief disorder instrument for Portuguese population. Article in Portuguese. Acta Med Port.

[42] (2020). Velórios sem padre e funerais limitados a dez pessoas. Como a Covid-19 está a mudar as cerimónias fúnebres em Portugal. Article in Portuguese. Observador.

[43] (2020). Presidência do Conselho de Ministros Decreto no. 2-B/2020. Article in Portuguese. Diário da República.

[44] (2012). Está a ser vítima? Article in Portuguese. Associação Portuguesa de Apoio à Vitima (APAV).

[45] Coleman J (2009). Relational analysis: the study of social organizations with survey methods. Human Organization.

[46] Goodman R, Kish L (1950). Controlled selection--a technique in probability sampling. J Am Stat Assoc.

[47] Atkinson R, Flint J (2001). Accessing hidden and hard-to-reach populations: Snowball research strategies. Social Research Update.

[48] Brickman-Bhutta C (2011). Not by the Book: Facebook as Sampling Frame. Sociological Methods & Research.

[49] Maia I, Monjardino T, Frias B, Canhão H, Cunha Branco J, Lucas R, Santos AC (2019). Food insecurity in Portugal among middle- and older-aged adults at a time of economic crisis recovery: prevalence and determinants. Food Nutr Bull.

[50] Maia I, Monjardino T, Lucas R, Ramos E, Santos AC (2019). Household food insecurity and socio-demographic determinants in young adults: findings from a Portuguese population-based sample. Int J Public Health.

[51] Traat I (2000). Theory of Sample Surveys. Statist Med.

[52] Downe-Wamboldt B (1992). Content analysis: method, applications, and issues. Health Care Women Int.

[53] Braun V, Clarke V (2006). Using thematic analysis in psychology. Qual Res Psychol.

[54] Wisdom J, Creswell JW (2013). Mixed Methods: Integrating Quantitative and Qualitative Data Collection and Analysis While Studying Patient-Centered Medical Home Models.

[55] de Oliveira AM, Buchain PC, Vizzotto ADB, Elkis H, Cordeiro Q, Gellman MD, Turner JR (2013). Psychosocial Impact. Encyclopedia of Behavioral Medicine.

[56] Wang C, Pan R, Wan X, Tan Y, Xu L, Ho CS, Ho RC (2020). Immediate psychological responses and associated factors during the initial stage of the 2019 coronavirus disease (COVID-19) epidemic among the general population in China. Int J Environ Res Public Health.

[57] Zandifar A, Badrfam R (2020). Iranian mental health during the COVID-19 epidemic. Asian J Psychiatr.

