# Supplementary material for: Psychological Impact of the COVID-19 Pandemic and Social Determinants on the Portuguese Population: Protocol for a Web-Based Cross-sectional Study
Source: JMIR Res Protoc. 2021 Oct 19;10(10):e28071. doi: 10.2196/28071 (PMC8528386; doi:10.2196/28071)
Supplement: Multimedia Appendix 1 [file resprot_v10i10e28071_app1.docx]

*Appendix 1. Questionnaire*

**Information to participants**

This questionnaire is part of the PhD project in Public Health of the researcher Ana Aguiar, affiliated within the Institute of Public Health of the University of Porto, and is entitled “Psychological impact and socio-environmental determinants on a Portuguese population during and after the pandemic by COVID-19” (Study funded by the Foundation for Science and Technology).

This project aims:

1) to study the psychological impact of the COVID-19 pandemic and the respective containment measures, as well as the individual and socio-environmental determinants of mental health during this crisis; and,

2) to analyze possible behavioral changes in the population during and after the pandemic period regarding the (ab) use of psychoactive substances, namely in their relationship with the informal economy; the dynamics associated with processes of domestic violence and social discrimination, unemployment, grieving issues, and food insecurity;

3) inclusion criteria: be at least 18 years old.

With regard to the methodology, this questionnaire is based on “snowball sampling”, that is, a non-probabilistic technique where the selected individuals invite new participants to participate within their network of friends and acquaintances. The name "snowball" comes from this idea: as a snowball goes down a slope, its size increases. The same happens in this sampling technique - it grows as the selected people invite new participants.

The questions will be the same for all participants, and you are not required to answer all questions.

Anything you tell us is confidential and anonymous. We do not collect any personal or contact information that allows you to be identified. However, as a rule, for the information to be collected, it is necessary to explicitly accept participation in the study (you can do it by clicking on the button that appears below) and your authorization for the collection of sociodemographic data (even if anonymized).

Your participation is valuable to know the impact of the pandemic and to create/adapt the necessary responses. Thank you very much for your participation.

**Informed consent**

I declare that I am over 18 years of age and I declare that I have read and understood the information presented above. I was guaranteed the possibility, at any point in the questionnaire, to refuse to answer some questions in this study without any kind of consequences. In this way, I accept to participate in this study and allow the use of the data that I voluntarily provide, trusting that they will only be used for this investigation and in the guarantees of confidentiality and anonymity that are given to me by the researcher.

☐ I agree to participate in the study

☐ I don't want to participate in the study

**Sociodemographic characteristics**

**1.** Date of birth: ____/____/___ (dd/mm/yy)

**2.** Gender:

☐ Male

☐ Female

☐ Other, which _______________

**3.** What is the highest level of education you completed:

☐ Can't read or write

☐ Can read and write but has not completed the 1st cycle of schooling

☐ 1st cycle of basic education (4th year)

☐ 2nd cycle of basic education or lower (6th year)

☐ 3rd cycle of education (9th year)

☐ Secondary education (12th grade)

☐ Bachelor/Degree

☐ Master or PhD

☐ Other, which ______________________________

☐ Don't want to answer

**4.** Residence council: ___________________________

**5.** Marital status:

☐ Married / de facto union

☐ Single

☐ Divorced / separated

☐ Widower

☐ Don't want to answer

**6.** Professional situation on 1^st^ January 2020:

☐ Student

☐ In active (specify profession): ______________________

☐ Household

☐ Retired

☐ Unemployed

☐ Other (specify): ___________

☐ Don't want to answer

**6.1.** Has your professional situation changed since the beginning of the pandemic by COVID-19 and as a direct consequence of the pandemic?

☐ Yes (if yes, go to question 6.1.1)

☐ No (if not, go to question 7)

**6.1.1**. How do you rate this change in terms of the impact it has had on your life? Please use the scale below to select the option that best suits you, with 1 referring to “extremely negative” and 5 referring to “extremely positive”:

| 1  Extremely negative | 2 | 3 | 4 | 5 | 6  Extremely positive |
| --- | --- | --- | --- | --- | --- |
| ☐ | ☐ | ☐ | ☐ | ☐ | ☐ |

**6.1.2.** Please indicate the extent to which your professional situation has changed since March 16, 2020 to the present:

☐ Continued to work in person

☐ Started working in telework

☐ Was unemployed before and during the COVID-19 outbreak

☐ Was unemployed during the current COVID-19 pandemic emergency

☐ Household

☐ Retired

☐ Other (specify): ___________

☐ Don't want to answer

**7.** How do you consider your household income?

☐ Insufficient

☐ You have to be careful about spending

☐ Enough for your needs

☐ Comfortable

☐ Don't want to answer

**7.1.** Has your household's income changed due to the pandemic?

☐ Yes, have been reduced

☐ Yes, they have been increased

☐ There was no change

☐ Don't want to answer

**7.2.** Household (number of people including yourself): ______________

**Children and teleschool**

**8.** Are there dependents in your household?

(for a dependent person, consider the following definitions: a person who does not have his own means of subsistence, living at the expense of others; a person who has a physical and / or psychological need for a particular substance or activity).

☐ Yes (if yes, go to question 8.1.1)

☐ No (if not, go to question 9)

☐ Do not want to answer (if you do not want to answer, go to question 9)

**8.1.1.** How many are dependents of legal age (≥18 years)? _______

**8.1.2.** How many are underage dependents (<18 years)? ______

**8.2.** Who does the dependent person live with (select all that apply)?

|  | Live with |
| --- | --- |
| Mother | ☐ |
| Father | ☐ |
| Companion of the Mother other than the Father | ☐ |
| Companion of the Father other than the Mother | ☐ |
| Maternal grandparents | ☐ |
| Paternal grandparents | ☐ |
| Brothers | ☐ |
| Half brothers | ☐ |
| Other family members | ☐ |

**8.2.1.** Are any dependents studying?

☐ Yes (if yes, go to question 8.2.2)

☐ No (if you do not proceed to question 9)

**8.2.2.** Since schools closed and mandatory confinement (applied between March 13th and May 4th), have your children taken classes from home?

☐ Yes (if yes, go to question 8.2.1.1)

☐ No

**8.2.2.1.** Did the fact that your children were forced to stay at home and take distance classes cause any change in your routine? If so, please describe how it has positively and / or negatively influenced your personal and professional life:

____________________________________________________________

____________________________________________________________

**8.2.2.2.** At this moment, after November 4, and taking into account the renewed situation of public calamity and subsequent state of emergency in national territory, your children:

☐ They are taking face-to-face classes since the beginning of the school year

☐ They were in face-to-face classes, but right now they are taking classes at home

☐ They have been on a telescope since the beginning of the school year

☐ Another situation

[Other] Please describe the situation:

_______________________________________________________________

_________________________________________________________

**8.3.** Do you want to add any information regarding children and teleschool? If so, please leave your comments, observations, feelings or doubts in the text field below:

___________________________________________________________

___________________________________________________________

**Behaviors and habits**

**Use of psychoactive substances**

**9.** Do you smoke or have you ever smoked?

☐ Yes

☐ No (if you do not proceed to question 10)

☐ Don't want to answer

**9.1.** On average how many cigarettes do you smoke per day? ______

**9.2.** Have there been any changes in your smoking habits since the beginning of the pandemic? Please select the option that best suits you:

☐ Yes, I reduced my cigarette consumption

☐ Yes, I increased my cigarette consumption

☐ No, I continued to smoke the same number of cigarettes

☐ Don't want to answer

**10.** Do you drink or have you ever drank alcoholic beverages?

☐ Yes

☐ No (if you do not proceed to question 11)

☐ Don't want to answer

**10.1.** In the past month, how often did you drink alcohol-containing drinks?

| Average frequency | | | | | | | | |
| --- | --- | --- | --- | --- | --- | --- | --- | --- |
| Never or <1 month | 1-3 per month | 1 per week | 2-4 per week | 5-6 per week | 1 a day | 2-3 per day | 4-5 per day | 6 or more per day |
| ☐ | ☐ | ☐ | ☐ | ☐ | ☐ | ☐ | ☐ | ☐ |

**10.2.** [If yes in question 10], since the beginning of the pandemic:

☐ Average consumption of alcoholic beverages increased

☐ Decreased the average frequency of consumption of alcoholic beverages

☐ Maintained the average frequency of alcohol consumption

☐ Don't want to answer

**11.** Have you ever used illicit drugs? (including cannabis)

☐ Yes

☐ No (if you do not proceed to question 12)

☐ Don't want to answer

**11.1.** In which way did you use drugs?

☐ Injected

☐ Smoked

☐ Inhaled / sniffed

☐ Don't want to answer

☐ Other (s): ________________________________

**11.2.** When was the last time you used it?

☐ In the last 30 days

☐ In the last 12 months

☐ More than 12 months ago

☐ Don't want to answer

**11.3.** What kind of substances do you normally consume and in which way?

|  | ***Smoked*** | ***Injected*** | ***Inhaled*** | ***Other*** |
| --- | --- | --- | --- | --- |
| Cannabis | ☐ | ☐ | ☐ | ☐ |
| Cocaine | ☐ | ☐ | ☐ | ☐ |
| Heroin | ☐ | ☐ | ☐ | ☐ |
| Ecstasy | ☐ | ☐ | ☐ | ☐ |
| Amphetamines | ☐ | ☐ | ☐ | ☐ |
| LSD | ☐ | ☐ | ☐ | ☐ |
| Another | ☐ | ☐ | ☐ | ☐ |

☐ Don't want to answer

**11.4.** Approximately how long ago (years) did consumption start? _________________

**11.5.** [If “in the last 30 days” or “in the last 12 months” in question 11.2], since the start of the pandemic:

☐ Median average frequency of consumption of illicit drugs increased

☐ Decreased the median average frequency of consumption of illicit drugs

☐ Maintained the average frequency of consumption of illicit drugs

☐ Don't want to answer

**Behaviours and habits**

**Food**

**Household Food Security** *[U.S. Household Food Security Survey Module: Six-Item Short Form. Economic Research Service, USDA]*

The following statements refer to phrases that people have referred to to describe their situation regarding food. For these statements, please tell me whether the statement was often true, sometimes true, or never true for (you/your household) in the last 12 months.

**12.** “The food that (I/we) bought just didn’t last, and (I/we) didn’t have money to get more.” Was that often, sometimes, or never true for (you/your household) in the last 12 months?

☐ Often true

☐ Sometimes true

☐ Never true

☐ Dont know

☐ Refuse to answer

**13.** “(I/we) couldn’t afford to eat balanced meals.” Was that often, sometimes, or never true for (you/your household) in the last 12 months?

☐ Often true

☐ Sometimes true

☐ Never true

☐ Dont know

☐ Refuse to answer

**14.** In the last 12 months, since last, did (you/you or other adults in your household) ever cut the size of your meals or skip meals because there wasn't enough money for food?

☐ Yes (advance to question 14.1)

☐ No (advance to question 15)

☐ Dont know (advance to question 15)

☐ Refuse to answer (advance to question 15)

**14.1.** [If yes above] How often did this happen—almost every month, some months but not every month, or in only 1 or 2 months?

☐ Almost every month

☐ Some months but not every month

☐ Only 1 or 2 months

☐ Dont know

☐ Refuse to answer

**15.** In the last 12 months, did you ever eat less than you felt you should because there wasn't enough money for food?

☐ Yes

☐ No

☐ Dont know

☐ Refuse to answer

**16.** In the last 12 months, were you every hungry but didn't eat because there wasn't enough money for food?

☐ Yes

☐ No

☐ Dont know

☐ Refuse to answer

**17.** Regarding questions 12 to 16, did these situations worsen during the pandemic?

☐ Yes

☐ No

☐ Not applicable

**17.1.** [if yes] could you please describe how they got worse and what impact this had on your household?

_____________________________________________________________________

_____________________________________________________________________

**Clinical background and health care**

**18.** Do you currently have a condition that requires regular health care (a disease diagnosed by your doctor)?

☐ Yes

☐ No (advance to question 19)

**18.1.** [if yes] Select from the list below all that apply:

| Disease | Yes |
| --- | --- |
| Arterial hypertension | ☐ |
| Diabetes | ☐ |
| High cholesterol | ☐ |
| Asthma | ☐ |
| Chronic bronchitis | ☐ |
| Chronic obstructive pulmonary disease | ☐ |
| Cancer | ☐ |
| Autoimmune disease | ☐ |
| Epilepsy | ☐ |
| Depression | ☐ |
| Other (which): __________________ | ☐ |
| Other (which): __________________ | ☐ |
| Other (which): __________________ | ☐ |
| Other (which): __________________ | ☐ |

**19.** Do you usually take anxiolytics and antidepressants?

☐ Yes

☐ No (if you do not proceed to question 20)

☐ Don't want to answer

**19.1.** [if yes] Please indicate the medication you are taking and what health problem you are referring to:

| Medication | Health problem |
| --- | --- |
|  |  |
|  |  |
|  |  |
|  |  |
|  |  |
|  |  |

**19.2.** [if you usually take anxiolytics and antidepressants] did you take it before the pandemic?

☐ Yes (go to question 20)

☐ No (go to question 19.2.1)

☐ Don't want to answer

**19.2.1.** [if not] can you please indicate the reason for having started taking the medication during the COVID-19 pandemic?

____________________________________________________________

____________________________________________________________

____________________________________________________________

**Infection with the new coronavirus (SARS-CoV-2)**

**20.** From your knowledge, since March 2020 have you contacted someone who has been diagnosed with the infection?

☐ Yes

☐ No

**20.1.** In what month was that contact?

| March | April | May | June | July | August | Sept | Oct | Nov | Dec |
| --- | --- | --- | --- | --- | --- | --- | --- | --- | --- |
| ☐ | ☐ | ☐ | ☐ | ☐ | ☐ | ☐ | ☐ | ☐ | ☐ |

**21.** Since the beginning of March, have you had any of the following situations? Please select all that apply:

☐ I had a confirmed diagnosis of SARS-CoV-2 infection (go to question 22)

☐ The person(s) living with me had had a positive diagnosis

☐ I was in prophylactic isolation for having contacted a positive person

☐ Other (please describe which):__________________________________________

**21.1.** What impact did this situation (s) have on the essential dimensions from the point of view of mental health (for example: anxiety, depression). Please describe in the text field below:

_____________________________________________________________________

_____________________________________________________________________

[if diagnosis confirmed]

**21.2.** How long have you been positive? _______________ (days or months)

**21.3.** [if diagnosis confirmed] please indicate the symptoms you had:

| **Symptoms** |  | |
| --- | --- | --- |
| Persistent cough | ☐ Yes | ☐ No |
| Respiratory difficulties | ☐ Yes | ☐ No |
| Nasal discharge | ☐ Yes | ☐ No |
| Sore throat | ☐ Yes | ☐ No |
| Chest pain | ☐ Yes | ☐ No |
| Abdominal pain | ☐ Yes | ☐ No |
| Vomiting / Nausea | ☐ Yes | ☐ No |
| Diarrhea | ☐ Yes | ☐ No |
| Fever (> 37.5ºC) | ☐ Yes | ☐ No |
| Headache | ☐ Yes | ☐ No |
| Joint pain | ☐ Yes | ☐ No |
| Muscle pain throughout the body | ☐ Yes | ☐ No |
| Reduced sense of smell | ☐ Yes | ☐ No |
| Decreased taste | ☐ Yes | ☐ No |
| General weakness or asthenia | ☐ Yes | ☐ No |
| Other (which): _________________________________ | ☐ Yes | ☐ No |
| Other (which): _________________________________ | ☐ Yes | ☐ No |

**Additional information on COVID-19**

If you have one or more of the above symptoms you should contact SNS 24 - 808242424 or in an emergency 112.

**Mental Health**

**Hospital Anxiety and Depression Scale (HADS)** *[Escala de Depressão e Ansiedade (HADS), portuguese version used from Pais-Ribeiro et al., 2007]*

**22.** Following, a set of statements will be presented on how you have been feeling in the last week. Please indicate the one that most closely matches the way you felt (check only one answer for each paragraph):

**22.1.** I feel tense or 'wound up':

☐ Most of the time

☐ A lot of time

☐ From time to time, occasionally

☐ Not at all

**22.2.** I still enjoy the things I used to enjoy:

☐ Definitely as much

☐ Not quite so much

☐ Only a little

☐ Hardly at all

**22.3.** I get a sort of frightened feeling as if something awful is about to happen:

☐ Very definitely and quite badly

☐ Yes, but not too badly

☐ A little, but it doens’t worry me

☐ Not at all

**22.4.** I can laugh and see the funny side of things:

☐ As much as I always could

☐ Not quite so much now

☐ Definitely not so much now

☐ Not at all

**22.5.** Worrying thoughts go through my mind:

☐ A great deal of the time

☐ A lot of time

☐ From time to time, but not too often

☐ Only occasionally

**22.6.** I feel cheerful:

☐ Not at all

☐ Not often

☐ Sometimes

☐ Most of the time

**22.7.** I can sit at ease and feel relaxed:

☐ Definitely

☐ Usually

☐ Not often

☐ Not at all

**22.8.** I feel as if I am slowed down:

☐ Nearly all the time

☐ Very often

☐ Sometimes

☐ Not at all

**22.9.** I get a sort of frightened feeling like 'butterflies' in the stomach:

☐ Not at all

☐ Occasionally

☐ Quite often

☐ Very often

**22.10.** I have lost interest in my appearance:

☐ Definitely

☐ I don’t take as much care as I should

☐ I may not take quite as much care

☐ I take just as much care as ever

**22.11.** I feel restless as I have to be on the move:

☐ Very much indeed

☐ Quite a lot

☐ Not very much

☐ Not at all

**22.12.** I look forward with enjoyment to things:

☐ As much as I ever did

☐ Rather less than I used to

☐ Definitely less than I used to

☐ Hardly at all

**22.13.** I get sudden feelings of panic:

☐ Very often indeed

☐ Quite often

☐ Not very often

☐ Not at all

**22.14.** I can enjoy a good book or radio or TV program:

☐ Often

☐ Sometimes

☐ Not often

☐ Very seldom

**23.** Taking into account the previous statements and their responses, to what extent do you relate what you described to the pandemic? Please describe the relationship in the text field below: ________________________________________________________________

______________________________________________________________________

______________________________________________________________________

**23.1**. If you said yes to any of the previous statements, was any of these situations related to the infection? Please select from the scale below the option that best suits you:

| 1  Nothing related | 2  Amlittle related | 3  Related | 4  Very related | 5  Very much related |
| --- | --- | --- | --- | --- |
| ☐ | ☐ | ☐ | ☐ | ☐ |

**Additional information on COVID-19**

The current COVID-19 pandemic has led to profound changes in people's lives. If you feel some type of psychological pressure that is difficult to control, or if you need support for another type of situation, you should call the NHS 24 - 808242424 and select option 4 (psychological counseling). The service is available 24 hours a day, 7 days a week. No calls are recorded and the service is performed by clinical psychologists.

**Mourning**

**Mourning** *[PG-13 – Prolonged Grief Disorder, portuguese version from Delalibera M et al., 2010]*

**23.** Then we will present some questions about the grieving process.

**23.1.** Since the beginning of the COVID-19 pandemic, has anyone lost / died that you consider significant?

☐ Yes

☐ No (if you don't move on to violence issues)

☐ Do not want to answer (if you do not want to answer, advance to questions about violence)

**23.2.** How long ago did this happen? ________

**23.3.** What was the bond you had with this person? (for example: mother, grandfather, ...) ________________________________

**23.4.** How emotionally difficult was this loss for you? Use the scale below to answer that 1 is "emotionally bearable" and 6 "emotionally unbearable"

| 1  emotionally bearable | 2 | 3 | 4 | 5 | 6  emotionally unbearable |
| --- | --- | --- | --- | --- | --- |
| ☐ | ☐ | ☐ | ☐ | ☐ | ☐ |

**24.** Tick your answer for each item:

**24.1.** In the last month, how often have you felt longing or yearning for the person you lost?

☐ Almost never

☐ At least once

☐ At least once a week

☐ At least once a day

☐ Several times a day

**24.2.** In the last month, how often have you had intense feelings of emotional pain, sorrow or pangs of grief?

☐ Almost never

☐ At least once

☐ At least once a week

☐ At least once a day

☐ Several times a day

**24.3.** With regard to questions 24.1 and 24.2, have you had this experience at least daily, for a period of at least 6 months?

☐ Yes

☐ No

**24.4.** In the last month, how often have you tried to avoid reminders that the person you have los tis gone?

☐ Almost never

☐ At least once

☐ At least once a week

☐ At least once a day

☐ Several times a day

**24.5.** In the last month, how often have you felt stunned, schoed, or dazed by your loss?

☐ Almost never

☐ At least once

☐ At least once a week

☐ At least once a day

☐ Several times a day

**25.** Following, a set of statements is presented regarding your loss. For each of the statements, please indicate how you usually feel using the following scale: 1 “No, not at all”, 2 “Slightly”, 3 “Reasonably”, 4 “Enough” and 5 “Extremely”.

| Q | Affirmation | 1 | 2 | 3 | 4 | 5 |
| --- | --- | --- | --- | --- | --- | --- |
| 1 | Do you feel confused about your role in life or do you feel you don't know who you are (i.e., do you feel that a part of you has died)? | ☐ | ☐ | ☐ | ☐ | ☐ |
| 2 | Have you had difficulty accepting the loss? | ☐ | ☐ | ☐ | ☐ | ☐ |
| 3 | Have you had difficulty trusting others since the loss? | ☐ | ☐ | ☐ | ☐ | ☐ |
| 4 | Do you feel bitterness for your loss? | ☐ | ☐ | ☐ | ☐ | ☐ |
| 5 | Do you now find it difficult to get on with your life (for example, making new friends, having new interests)? | ☐ | ☐ | ☐ | ☐ | ☐ |
| 6 | Do you feel emotionally numb since your loss? | ☐ | ☐ | ☐ | ☐ | ☐ |
| 7 | Do you feel that your life is unsatisfactory, empty or meaningless since your loss? | ☐ | ☐ | ☐ | ☐ | ☐ |

|  |  |  |  |  |  |
| --- | --- | --- | --- | --- | --- |

**25.1**. To what extent did your loss impact your day-to-day life? Please describe in the text field below: _______________________________________________________________

____________________________________________________________________________

____________________________________________________________________________

**Violence**

**23.** Dating Violence / Love Relationship Questionnaire *[Portuguese Association for Victim Support (APAV) Domestic Violence Questionnaire]*

**23.1.** Have you been in a loving relationship since January 2020?

☐ Yes

☐ No (end of questionnaire)

**23.1.1.** [if yes] We would like to know how many times you have experienced each of the following behaviors in your intimate relationship since January 2020. Use the following scale to classify each of the situations presented, where: 0 corresponds to "never", 1 "rarely", 2 “sometimes”, 3 “often” and 4 “always”.

Select the option that best fits your experience:

| Q | Affirmation | 0 | 1 | 2 | 3 | 4 |
| --- | --- | --- | --- | --- | --- | --- |
| 1 | “Test” your love, set traps to find out if you are cheating | ☐ | ☐ | ☐ | ☐ | ☐ |
| 2 | You feel compelled to have sex, as long as you don't have to explain why | ☐ | ☐ | ☐ | ☐ | ☐ |
| 3 | He/She hit me | ☐ | ☐ | ☐ | ☐ | ☐ |
| 4 | He/She is always late for meetings, he does not deliver what he promises and he is irresponsible | ☐ | ☐ | ☐ | ☐ | ☐ |
| 5 | Talk to you about relationships he / she thinks you have | ☐ | ☐ | ☐ | ☐ | ☐ |
| 6 | Insists on touching you in ways and places where you don't like and don't want to | ☐ | ☐ | ☐ | ☐ | ☐ |
| 7 | He/She slapped you in the face, pushed you or shook you | ☐ | ☐ | ☐ | ☐ | ☐ |
| 8 | Does not recognize any responsibility for the relationship or what happens to the two | ☐ | ☐ | ☐ | ☐ | ☐ |
| 9 | Criticizes you, underestimates the person you are or humiliates your self-esteem | ☐ | ☐ | ☐ | ☐ | ☐ |
| 10 | Shot blunt objects (which can cause injury or bruising by pressure on a part of the body) at you | ☐ | ☐ | ☐ | ☐ | ☐ |
| 11 | Hurt you with an object | ☐ | ☐ | ☐ | ☐ | ☐ |
| 12 | Ridicules your way of expressing yourself | ☐ | ☐ | ☐ | ☐ | ☐ |
| 13 | Prevented you from leaving home (by physical means) | ☐ | ☐ | ☐ | ☐ | ☐ |
| 14 | Feels forced to perform certain sexual acts | ☐ | ☐ | ☐ | ☐ | ☐ |
| 15 | Ignored your feelings | ☐ | ☐ | ☐ | ☐ | ☐ |
| 16 | Stop talking to you or disappear for a few days, without any explanation, in order to show that he/she is bothered | ☐ | ☐ | ☐ | ☐ | ☐ |
| 17 | Invades your space (listening to loud music when studying / working, listening to your phone calls, etc.) | ☐ | ☐ | ☐ | ☐ | ☐ |
| 18 | Forces you to undress, even if you don't want to | ☐ | ☐ | ☐ | ☐ | ☐ |
| 19 | Ridiculed or insulted your beliefs, religion or social class | ☐ | ☐ | ☐ | ☐ | ☐ |
| 20 | Ridicules or insults you for the ideas that you defend | ☐ | ☐ | ☐ | ☐ | ☐ |

**23.** If you choose, sometimes, often or always selected in any of the previous questions, did these situations already occur before the COVID-19 pandemic began? Or did they only start after the contingency measures implemented in the country?

☐ Yes, it happened before the COVID-19 pandemic started

☐ No, it started during the COVID-19 pandemic

☐ Don't want to answer

**Additional information on Violence**

The presence of one or more of these behaviors, mainly used to control other people, can mean that you are a victim of physical, psychological or sexual violence in your relationship. Domestic violence is a crime. If you need help contact APAV: 116006 Victim support line; 800219090 Secure internet line.

**25.** Observations - we would like you to finally leave us your recommendations / ideas to: 1) take better care of mental health in this pandemic phase; 2) what services you know and what services need to be created and / or improved; 3) what would you recommend to politicians who make decisions? __________________________________________________________________

___________________________________________________________________________

___________________________________________________________________________

The questionnaire came to an end.

Thank you very much for your participation.

We now ask you to please send the questionnaire to 5 people in your network of contacts so that they can answer the questionnaire and allow us to reach a larger number of participants.
